# R. S. Fowler

**Published:** 1906-12

**Authors:** 


### R. S. FOWLER, F.R.C.S.,
### Bath.

We regret to find that the long illness of Mr. Richard Sumner Fowler, of Belmont, Bath, terminated in his decease on December 9th. An old student and associate of King's College, London, he became qualified in 1852, and has occupied a distinguished position in the medical life of the city of Bath from that time until his death, at the age of 76. In 1867 he became surgeon to the Bath United Hospital, but resigned that honorary appointment in 1890, and in 1891 he was elected one of the committee, in 1895 a trustee, and on the death of the Rev. E. Handley, in 1904, he became president of the institution. For many years he was secretary, and in 1884 he was elected president of the Bath and Bristol Branch of the British Medical Association, and he acted for a long period as secretary of the branch on the General Council. Mr. Fowler was a most devoted son of the Church of England, and had served the office of churchwarden at St. Paul's for 32 years. In 1859 he was among the first to join the Volunteer movement, being an original member of the Bath Rifles.

Mr. Fowler was held in much esteem by his professional brethren, and he received many presentations in recognition of his invaluable work and services to his fellow-citizens.

